# A multistate competing risks framework for preconception prediction of pregnancy outcomes

**DOI:** 10.1186/s12874-022-01589-7

**Published:** 2022-05-30

**Authors:** Kaitlyn Cook, Neil J. Perkins, Enrique Schisterman, Sebastien Haneuse

**Affiliations:** 1 Department of Population Medicine, Harvard Medical School and Harvard Pilgrim Health Care Institute, Boston, MA US; 2 Epidemiology Branch, Division of Intramural Population Health Research, Eunice Kennedy Shriver National Institute of Child Health and Human Development, Bethesda, MD US; 3 Department of Biostatistics, Epidemiology, and Informatics, Perelman School of Medicine, University of Pennsylvania, Philadelphia, PA US; 4 Department of Biostatistics, Harvard T.H. Chan School of Public Health, Boston, MA US

**Keywords:** Competing risks, Discrete survival models, Missing data, Multinomial classification, Pregnancy, Risk prediction

## Abstract

**Background:**

Preconception pregnancy risk profiles—characterizing the likelihood that a pregnancy attempt results in a full-term birth, preterm birth, clinical pregnancy loss, or failure to conceive—can provide critical information during the early stages of a pregnancy attempt, when obstetricians are best positioned to intervene to improve the chances of successful conception and full-term live birth. Yet the task of constructing and validating risk assessment tools for this earlier intervention window is complicated by several statistical features: the final outcome of the pregnancy attempt is multinomial in nature, and it summarizes the results of two intermediate stages, conception and gestation, whose outcomes are subject to competing risks, measured on different time scales, and governed by different biological processes. In light of this complexity, existing pregnancy risk assessment tools largely focus on predicting a single adverse pregnancy outcome, and make these predictions at some later, post-conception time point.

**Methods:**

We reframe the individual pregnancy attempt as a multistate model comprised of two nested multinomial prediction tasks: one corresponding to conception and the other to the subsequent outcome of that pregnancy. We discuss the estimation of this model in the presence of multiple stages of outcome missingness and then introduce an inverse-probability-weighted Hypervolume Under the Manifold statistic to validate the resulting multivariate risk scores. Finally, we use data from the Effects of Aspirin in Gestation and Reproduction (EAGeR) trial to illustrate how this multistate competing risks framework might be utilized in practice to construct and validate a preconception pregnancy risk assessment tool.

**Results:**

In the EAGeR study population, the resulting risk profiles are able to meaningfully discriminate between the four pregnancy attempt outcomes of interest and represent a significant improvement over classification by random chance.

**Conclusions:**

As illustrated in our analysis of the EAGeR data, our proposed prediction framework expands the pregnancy risk assessment task in two key ways—by considering a broader array of pregnancy outcomes and by providing the predictions at an earlier, preconception intervention window—providing obstetricians and their patients with more information and opportunities to successfully guide pregnancy attempts.

## Background

### The role of risk prediction in preconception care

In obstetrics and gynecology, preconception care refers to a “set of interventions that aim to identify and modify biomedical, behavioral, or social risks to [a potentially child-bearing individual’s] health or pregnancy outcome,” with particular emphasis placed on those factors that must be intervened on prior to conception or early in the pregnancy [1]. Integral to these efforts is the initial preconception consultation, a pre-pregnancy check-up during which an obstetrician may collect baseline demographic and medical history data in order to better guide and inform the planned pregnancy attempt. These consultations thus serve an important dual purpose: they assist patients in preparing for an upcoming pregnancy, while also representing the first point at which clinicians might intervene to improve the likelihood of conception and an eventual full-term live birth by, for example recommending immediate initiation of assisted reproductive technology [2] or by identifying and reviewing modifiable risk factors of adverse pregnancy outcomes [3, 4].

To that end, accurate and personalized predictions of both the time to conception and the outcome of any subsequent pregnancy are particularly relevant to preconception care [1]. Yet current pregnancy outcome prediction tools are limited in several key dimensions: existing risk scores typically (i) focus on prediction among individuals with already viable pregnancies, using as predictors biomarker and other biomedical data that may be unavailable or difficult to measure at an initial preconception visit [5–7], and (ii) either collapse all pregnancy outcomes into a single adverse event indicator [8, 9] or focus on prediction of a single pregnancy outcome [10, 11]. In focusing on a binarized representation of the pregnancy attempt, these models ignore the complex interdependencies and competing risks that may exist between pregnancy outcomes. This improper accounting may, in turn, have consequences for estimation biases in, and the predictive accuracy of, these post-conception prediction models [12, 13].

The few obstetric risk prediction tools explicitly developed for preconception contexts (such as those in Sep et al. [14], van Kuijk et al. [15], and Mehta-Lee et al. [16]) similarly consider only a single isolated adverse maternal or neonatal event, and are further limited by concerns of both internal and external validity. In particular, these models are often constructed and trained on retrospectively ascertained datasets whose inclusion criteria require that individuals (i) had a clinically-recognized pregnancy that (ii) resulted in a live birth during the course of the study period; such criteria systematically exclude individuals struggling with sub-fertility or clinical pregnancy loss—individuals who may also be at higher risk for other adverse pregnancy outcomes—while preventing the generalization of these models to practical preconception clinical settings in which neither conception nor an eventual live birth are guaranteed. And while greater attention has been paid to the task of modeling fertility and time to conception in the statistical literature [17–21], to the best of our knowledge no obstetric/gynecological model has attempted to integrate these fertility predictions into a broader preconception risk assessment framework.

In short, the existing pregnancy-related prediction tools are either all adapted for patient populations and clinical scenarios that are defined downstream of the initial preconception visit or are too narrow in scope to be used to guide preconception care.

### The effects of aspirin in gestation and reproduction trial

To address this limitation, we aim to develop a clinical risk assessment tool that is implementable during the course of a pre-pregnancy check-up and that simultaneously considers both (i) the likelihood of a clinically-recognized pregnancy occurring and (ii) the likelihood of that pregnancy ending in either a full-term live birth or an adverse event. This prediction task effectively reframes the pregnancy attempt as a multistate competing risks process comprised of two stages—a conception stage resulting in an implementation event (or a lack thereof) and a gestation stage resulting in a final birth outcome—that represent fundamentally different biological processes and thus may be governed by fundamentally different sets of risk factors [22].

One of the few existing pregnancy studies with data on both of these outcome stages, as well as a rich set of potential predictors, is the Effects of Aspirin in Gestation and Reproduction (EAGeR) trial, a randomized controlled trial of the effects of low-dose preconception aspirin use on both the times to conception and the subsequent birth outcomes for women with at least one prior pregnancy loss [23]. The study enrolled 1228 women at the time of their initial preconception consultation, and then followed these women for one of four mutually exclusive pregnancy attempt outcomes:

1. *failure to conceive* within six menstrual cycles of the initial preconception visit;
2. *pregnancy resulting in a clinical pregnancy loss*, including both spontaneous abortion and stillbirth;
3. *pregnancy resulting in a preterm birth*, defined as a live birth occurring at or before 37 weeks’ gestation; or
4. *pregnancy resulting in a full-term birth*, defined as a live birth occurring after 37 week’s gestation.

By treating the initial preconception visit as the time origin from which women are then prospectively followed for an implantation event, the EAGeR dataset overcomes the left and right truncation issues typically noted in time to pregnancy studies [24]; by recording pregnancy attempt outcomes on all women—regardless of whether those women ultimately fail to conceive within the study period or experience a clinical pregnancy loss—the trial circumvents the selection and generalizability concerns endemic to other preconception datasets.

It does, however, present different statistical challenges to the construction and validation of a preconception prediction model. In light of the relatively short follow-up during the conception stage of the EAGeR study, as well as the complex biological restrictions that naturally exist on the support of the subsequent birth outcomes, correct specification of the transition intensities for the various pregnancy attempt outcomes is difficult. Furthermore, the time-to-event data on each stage of the pregnancy attempt were recorded with different levels of granularity; the gestation-related outcomes (namely clinical pregnancy loss, preterm birth, and full-term birth) were reported in terms of gestational age, a continuous measure of time since implantation, while the conception outcome was reported in terms of menstrual cycles since the preconception visit. This partial coarsening of the time axis further complicates the use of traditional multistate modeling analyses for the preconception prediction of pregnancy outcomes, as these analyses typically assume that the outcome process unfolds and is measured entirely in either continuous or discrete time [25]. Finally, during the conduct of the EAGeR trial, women were lost to follow-up at both stages of the outcomes process—prior to the observation of a clinically-recognized pregnancy, as well as prior to the observation of that pregnancy’s outcome—such that the missing data process was also multistage in nature. As a consequence, we must contend with multiple possible sources of estimation bias when constructing the final risk prediction model, both as a consequence of missingness in the pregnancy attempt outcomes themselves *and* as a consequence of missingness in the selection event for the second-stage outcomes.

### Outline

In what follows, we present a unified framework for the preconception prediction of pregnancy outcomes that adapts the traditional multistate competing risks model in order to accommodate the coarsening of the timescale information and the multistage nature of the missingness processes, while also circumventing concerns regarding biological plausibility. In the “Methods” section, we first introduce this prediction framework in greater detail, and then discuss its estimation and inference in the presence of complete data (“Complete data setting” section) as well as extensions to the missing at random setting (“Estimation and validation in the presence of outcome missingness” section). We also present an analysis of data from the EAGeR trial (“Preconception risk prediction in the EAGeR study population” section), illustrating how preconception risk prediction might be accomplished and utilized by clinicians in practice. The results of this analysis are given in the “Results” section, and we conclude with a brief discussion in the “Discussion” section and conclusions in the “Conclusion” section.

## Methods

### Prediction in a multistate competing risks framework

**Fig. 1:**
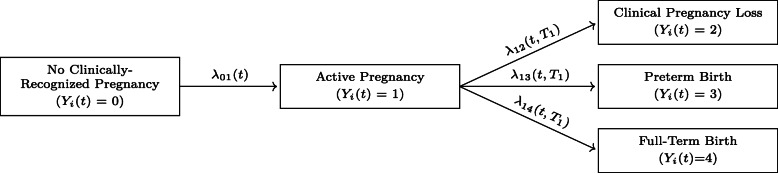
Multistate model characterizing the status of a single pregnancy attempt at calendar time *t* since the preconception visit, *Y*_*i*_(*t*). Arrows are labeled with the individual transition intensities

Suppose that individual *i* wishes to conceive, and schedules a preconception obstetrics consultation in order to plan their current pregnancy attempt. We let the stochastic process (*Y*_*i*_(*t*),*t*≥0) characterize the status of this attempt at time *t* since preconception visit, with *Y*_*i*_(*t*) taking on values over some discrete state space $\mathcal {S}$ that captures all potential pregnancy outcomes of interest. In the motivating EAGeR trial, for example, this state space is $\mathcal {S} = \{0, 1, 2, 3, 4\}$, where {0} indicates the absence of a successful implantation event, {1} an active and ongoing pregnancy, {2} a clinical pregnancy loss, {3} a preterm birth, and {4} a full-term birth. More complex constructions of $\mathcal {S}$ including additional terminal (e.g., elective abortion) and non-terminal (e.g., the development of gestational diabetes or pre-eclampsia) events are possible but not considered here. We assume that the transitions between these states are governed by the multistate model shown in Fig. 1, where

$$\lambda_{jk}(t) = {\lim}_{\delta \to 0} P\left\{Y_{i}(t^{-} + \delta) = k | Y_{i}(t^{-}) = j, \mathcal{Y}_{it^{-}}\right\} $$

characterizes the instantaneous probability of transitioning from pregnancy outcome state *j* to pregnancy outcome state *k* at time *t* since the preconception visit, conditional on the history of the pregnancy attempt up until that time, $\mathcal {Y}_{it^{-}}$. Note that the model in Fig. 1 naturally partitions the pregnancy attempt into a conception stage (the 0→1 transition) and a gestation stage (the 1→2,1→3, and 1→4 transitions); we assume that the transition intensity for the conception stage depends only on the time *t* since the preconception visit, and that the transition intensities for the gestation stage depend additionally on the time to clinically-recognized pregnancy, *T*_1_= inf{*t*:*Y*_*i*_(*t*)=1}. Given this framework, our aim is to make meaningful predictions about individual *i*’s pregnancy attempt, *Y*_*i*_(*t*)—and in particular to construct an individualized risk profile characterizing the likely outcomes of that attempt—based on the covariate information, ***w***_*i*_, that is routinely collected as part of the preconception consultation.

Several authors have considered how to incorporate covariate information into the estimation of the transition intensities, *λ*_*jk*_(*t*), and the state occupation probabilities, *π*_*ij*_(*t*)=*P*{*Y*_*i*_(*t*)=*j*}, of semi-Markovian stochastic processes like the multistate model in Fig. 1 (see Andersen and Perme [25] for an in-depth review). However, the nature of preconception pregnancy prediction in general—and the structure of the EAGeR data in particular—present several challenges to the construction of individual preconception risk profiles in terms of either of these quantities.

1. Directly specifying and estimating *λ*_*jk*_(*t*) in terms of ***w***_*i*_ and some parametric baseline intensity function, as discussed in Kalbfleisch and Prentice [26] and Beyersmann et al. [22] among others, is complicated by the complex restrictions that exist on the support of the gestational outcomes: clinical pregnancy loss, preterm birth, and full-term birth. The possible calendar times *t* at which *λ*_12_(*t*),*λ*_13_(*t*), and *λ*_14_(*t*) are each non-zero are dictated by limits on fetal viability, biological and medical constraints on pregnancy duration, and the definitions of the outcomes themselves, such that biologically plausible—let alone correct—specification of the baseline intensity functions is unlikely.
2. Alternatively, direct estimation of the state occupation probabilities using either the pseudo-value approach of Andersen and Klein [27] or the weighted estimating equation approach of Scheike and Zhang [28] and Scheike et al. [29] is complicated by the lack of granularity in the EAGeR event time data, and in pregnancy outcome reporting more generally. In particular, time to pregnancy is typically measured and reported in terms of menstrual cycles to conception, which partitions calendar time into *discrete* time units; the gestational outcomes (clinical pregnancy loss, preterm birth, and full-term birth) are instead reported in *continuous* time (gestational age) with an unknown and estimated time origin: the exact calendar time of conception. Using such data to estimate attributes of a single stochastic process (*Y*_*i*_(*t*),*t*≥0) with a cohesive and consistent internal time scale is challenging.
3. Similar data coarsening issues arise when accounting for censoring in the estimation task. In the EAGeR trial, for example, while exact loss to follow-up times are available for the conception stage of the stochastic process (i.e., for the 0→1 transition), the remaining pregnancy outcomes were determined using medical chart abstraction, so that only a binary indicator of missingness was available for the 1→2,1→3, and 1→4 transitions. The mechanisms driving missingess in the conception stage may also differ substantially from the mechanisms driving missingness in the clinical result of that conception.
4. Finally, while the multistate model for a single pregnancy attempt naturally includes a non-absorbing “active pregnancy” state (Fig. 1), this state is only of indirect interest for our particular prediction task. The relative likelihoods of occupying states {0}, corresponding to no clinically-recognized pregnancy; {2}, corresponding to clinical pregnancy loss; {3}, corresponding to preterm live birth; and {4}, corresponding to full-term live birth, have clear clinical and prognostic value for the pregnancy attempt. State {1}, corresponding to active pregnancy, is relevant only in so much as it defines the population of individuals on whom the gestation-related outcomes will be observed.

In light of these challenges, we instead define the individual preconception risk profile as

1 $$\begin{array}{*{20}l} p_{i}(\tau) :=& \left\{\pi_{i0}(\tau), \ \pi_{i2|1}(\tau)\pi_{i \neg 0}(\tau), \ \pi_{i3|1}(\tau)\pi_{i\neg 0}(\tau),\right. \\ &\left.\pi_{i4|1}(\tau)\pi_{i\neg 0}(\tau)\right\}, \end{array} $$

where *π*_*i*¬0_(*τ*):=1−*π*_*i*0_(*τ*) and where

$$\pi_{ij|1}(\tau) := {\lim}_{t \to \infty} P\{Y_{i}(t) = j | T_{1} \leq \tau\}, \quad j = 2, 3, 4 $$

are limiting conditional state occupation probabilities defined over a conception window of *τ* menstrual cycles. *p*_*i*_(*τ*) thus characterizes the likelihood that individual *i* fails to conceive within *τ* menstrual cycles of the preconception consultation, or that they successfully conceive within that window and that the subsequent pregnancy results in either a clinical pregnancy loss, a preterm birth, or a full-term birth: *π*_*i*0_(*τ*),*π*_*i*2|1_(*τ*)*π*_*i*¬0_(*τ*),*π*_*i*3|1_(*τ*)*π*_*i*¬0_(*τ*), and *π*_*i*4|1_(*τ*)*π*_*i*¬0_(*τ*), respectively.

**Fig. 2:**
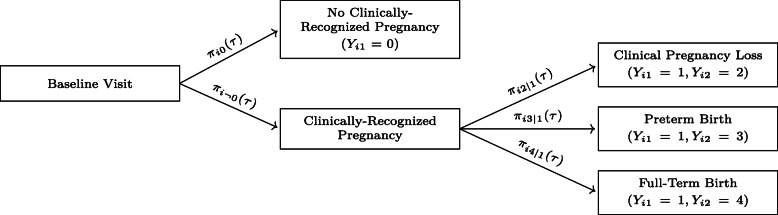
Reformulation of (*Y*_*i*_(*t*),*t*≥0) as a binomial first-stage outcome, *Y*_*i*1_, composed with a multinomial second-stage outcome, *Y*_*i*2_, conditional on a conception window of *τ* menstrual cycles. Arrows are labeled with the relevant individual (conditional) state occupation probabilities

Constructing *p*_*i*_(*τ*) in this fashion allows us to reformulate (*Y*_*i*_(*t*),*t*≥0) as the composition of a first-stage outcome, *Y*_*i*1_∈{0,1}, which represents conception within *τ* menstrual cycles, and a (potentially undefined) second-stage outcome, *Y*_*i*2_∈{2,3,4}, which represents the final result of that pregnancy (Fig. 2). We may then estimate *p*_*i*_(*τ*) in terms of the baseline covariate information ***w***_*i*_ by separately modeling the outcomes of each of these stages; this two-stage approach is reminiscent of the nested competing risks construction of [22] and allows us to explicitly account for the different timescales, the different governing risk factors, and the different censoring patterns of the conception and gestation processes while avoiding concerns regarding the biological plausibility of the underlying transition intensity model.

### Model estimation and validation

#### Complete data setting

When the reformulated pregnancy attempt outcome ***Y***_*i*_=(*Y*_*i*1_,*Y*_*i*2_) is completely observed for all *n* individuals, we take

2 $$\begin{array}{*{20}l} \text{logit}\left\{P(Y_{i1} = 1 | \boldsymbol{x}_{i})\right\} &= \boldsymbol{\beta}^{T}\boldsymbol{x}_{i}  \end{array} $$

3 $$\begin{array}{*{20}l} \log\left\{\frac{P(Y_{i2} = k | \boldsymbol{z}_{i}, Y_{i1} = 1)}{P(Y_{i2} = 4 | \boldsymbol{z}_{i}, Y_{i1} = 1)}\right\} &= \boldsymbol{\alpha}_{k}^{T}\boldsymbol{z}_{i}, \quad k=2, 3  \end{array} $$

where ***x***_*i*_⊂***w***_*i*_ and ***z***_*i*_⊂***w***_*i*_ are the baseline covariates informing the conception and gestation processes, respectively, and where the first element of ***x***_*i*_ and ***z***_*i*_ is assumed to be a 1 if an intercept is included. Note that the dependence of models (2) and (3) on the conception window *τ* is implicit in the definition of *Y*_*i*1_=*I*(*T*_1*i*_≤*τ*): different choices of *τ* will naturally lead to different definitions of the outcome for model (2) and the selection event for model (3), which might in turn lead to differences in the true values of ***β*** and ***α***. We further note that the second-stage model in (3) is agnostic to the timing of the clinically-recognized pregnancy beyond the implicit restriction that *Y*_*i*1_=1 ⇔ *T*_1*i*_≤*τ*; more complex specifications of (3), in which we first predict a time to conception and then incorporate this prediction into (3), are possible but not explored here. We then use standard maximum likelihood estimation to fit (2) to the full sample of *n* individuals and (3) to the subset of individuals with at least one clinically-recognized pregnancy within *τ* menstrual cycles of their preconception visit. From the resulting estimates of ***β***,***α***_2_, and ***α***_3_, we have

$$\begin{array}{*{20}l} \widehat{\pi}_{i\neg 0}(\tau) &= \text{expit}\left(\widehat{\boldsymbol{\beta}}^{T}\boldsymbol{x}_{i}\right)\\ \widehat{\pi}_{i2|1}(\tau) &= \widehat{\pi}_{i4|1}(\tau)\exp\left(\widehat{\boldsymbol{\alpha}}_{2}^{T}\boldsymbol{z}_{i}\right)\\ \widehat{\pi}_{i3|1}(\tau) &= \widehat{\pi}_{i4|1}(\tau)\exp\left(\widehat{\boldsymbol{\alpha}}_{3}^{T}\boldsymbol{z}_{i}\right) \\ \widehat{\pi}_{i4|1}(\tau) &= \left\{1 + \exp\left(\widehat{\boldsymbol{\alpha}}_{2}^{T}\boldsymbol{z}_{i}\right) + \exp\left(\widehat{\boldsymbol{\alpha}}_{3}^{T}\boldsymbol{z}_{i}\right)\right\}^{-1}, \end{array} $$

and we obtain the predicted risk profile for individual *i*, $\widehat {p}_{i}(\tau)$, by substituting these estimates into (1).

To quantify the extent to which these risk profiles meaningfully discriminate between the four pregnancy outcomes, we use the Hypervolume Under the Manifold (HUM) statistic, a generalization of the Area Under the Curve (AUC) statistic to outcomes with *K*>2 outcome classes. Much as the AUC measures the correct classification rate of discordant pairs of binary outcomes, the HUM measures the correct classification rate of sets of *K* individuals, with one individual from each of the *K* outcome classes. Operationalizing the HUM thus requires specifying a decision rule with which to conduct this *K*-alternative forced-choice decision task. To that end, let ***e***_*k*_ be the *k*th basis vector, here indicating assignment to outcome class *k* (*k*=1,…,*K*). We consider the following general class of decision rules, which selects the classification (*c*_1_,*c*_2_,…,*c*_*K*_) that minimizes the weighted sum of the Euclidean distances from each individual’s risk profile to their assignment vector:

4 $$\begin{array}{*{20}l} \underset{(c_{1}, c_{2}, \ldots, c_{K})}{\text{argmin}}& \left(\omega_{1}\|p^{(1)}(\tau) - \boldsymbol{e}_{c_{1}}\| + \omega_{2}\|p^{(2)}(\tau) - \boldsymbol{e}_{c_{2}}\|\right. \\&\left.+ \cdots + \omega_{K} \| p^{(K)}(\tau) - \boldsymbol{e}_{c_{K}}\|\right), \end{array} $$

where *p*^(*k*)^(*τ*) is the risk profile for the individual from class *k* and where ∥·∥ is the standard *L*_2_ norm. Note that the choice of weight vector, ***ω***=(*ω*_1_,…,*ω*_*K*_), allows one to differentially prioritize the discriminatory ability of *p*_*i*_(*τ*) with respect to each of the different outcome classes; a weighting scheme in which *ω*_1_/∥***ω***∥>0.25 will, for example, result in an HUM that rewards risk profiles with high discriminatory ability with respect to the first outcome class. Appropriate selection of ***ω*** is thus highly context dependent. In the absence of any strong rationale to the contrary, we recommend implementing (4) with all outcomes weighted equally, i.e., with ***ω*** set to ***1***, so that the resulting HUM speaks to *overall* classification performance and may be more readily compared across analyses and applications.

Let *CR*(·) indicate whether all *K* individuals have been correctly classified on the basis of their risk profiles and the selected decision rule. Then

$$HUM = P\left[CR\left\{p^{(1)}(\tau), p^{(2)}(\tau), \ldots, p^{(K)}(\tau)\right\} = 1\right] $$

and a nonparametric estimator of the HUM is

5 $$\begin{array}{*{20}l} \widehat{HUM} &\,=\, \frac{1}{n_{1}n_{2}\cdots n_{K}}\sum_{i_{1}=1}^{n_{1}}\sum_{i_{2}=1}^{n_{2}} \!\cdots\! \sum_{i_{K}=1}^{n_{K}}\\ &\quad CR\left\{\widehat{p}_{i_{1}}^{(1)}(\tau), \widehat{p}_{i_{2}}^{(2)}(\tau), \ldots, \widehat{p}_{i_{K}}^{(K)}(\tau)\right\}, \end{array} $$

where *n*_*k*_ is the number of individuals belonging to outcome class *k* and $\widehat {p}_{i_{k}}^{(k)}(\tau)$ is the estimated risk profile for the *i*_*k*_th individual in that class. Note that if the risk profiles have no inherent predictive value, such that they correspond to classification by random chance, then the correct classification rate will simply be *HUM*=1/*K*!. For pregnancy outcome prediction with *K*=4 outcome classes, this non-informative *HUM*=1/4!≈0.0417.

#### Estimation and validation in the presence of outcome missingness

Longitudinal pregnancy outcome studies often, however, feature loss to follow-up and study withdrawal, which may occur at any point during the pregnancy attempt. As a result, ***Y***_*i*_=(*Y*_*i*1_,*Y*_*i*2_) may be partially or completely unobserved for some subset of individuals in the study. Let *R*_*i*1_,*R*_*i*2_, and *R*_*i*_ be indicators of non-missingness in *Y*_*i*1_,*Y*_*i*2_, and ***Y***_*i*_, respectively, where *R*_*i*_=*I*(*R*_*i*1_=1∩*R*_*i*2_=1). Then study subjects may be classified as belonging to one of four observed data categories:

- Censored prior to conception (*R*_*i*1_=0,*R*_*i*2_=0)
- No pregnancy within *τ* menstrual cycles (*R*_*i*1_=1,*R*_*i*2_=1)
- Pregnancy within *τ* menstrual cycles, unknown result (*R*_*i*1_=1,*R*_*i*2_=0)
- Pregnancy within *τ* menstrual cycles, known result (*R*_*i*1_=1,*R*_*i*2_=1)

For an individual to have complete data with respect to model (2), their pregnancy status after *τ* cycles must be known (*R*_*i*1_=1); for an individual to have complete data with respect to model (3), both their pregnancy status and, provided that a clinically-recognized pregnancy occurred, the final result of that pregnancy must be observed (*R*_*i*1_=1,*Y*_*i*1_=1,*R*_*i*2_=1).

Modeling and predicting ***Y***_*i*_ thus requires addressing potentially multiple stages of outcome missingness, where the factors governing that missingness may vary according to stage. To that end, we assume that ***Y***_*i*_ is missing sequentially at random, so that

$$\begin{array}{*{20}l} P(R_{i1}= 1 | \boldsymbol{w}_{i}, Y_{i1}) &= P\left(R_{i1}=1 | \boldsymbol{x}_{i}^{\prime}\right)\\ {}P(R_{i2}= 1 | \boldsymbol{w}_{i}, R_{i1}=1, \boldsymbol{Y}_{i}) &= P\left(R_{i2} = 1 | \boldsymbol{z}_{i}^{\prime}, R_{i1}=1, Y_{i1}\right)\\ P(R_{i}= 1| \boldsymbol{w}_{i}, \boldsymbol{Y}_{i}) &= P\left(R_{i2} = 1 | \boldsymbol{z}_{i}^{\prime}, R_{i1}=1, Y_{i1}\right)\\ &\quad\ P(R_{i1}=1 | \boldsymbol{x}_{i}'), \end{array} $$

where ***x****i*′⊂***w***_*i*_ and ***z****i*′⊂***w***_*i*_ are the baseline covariates informing the first and second stages of the missingness process, respectively. We then (i) reframe estimation of *π*_*i*¬0_(*τ*)=*P*(*Y*_*i*1_=1|***x***_*i*_) in terms of a discrete-time survival model for time to clinically-recognized pregnancy, and (ii) implement the modularized missing data framework of Haneuse and Daniels [30] using either multiple imputation or inverse probability of censoring weights to address selection into and estimation of the model for *π*_*ik*|1_(*τ*)=*P*(*Y*_*i*2_=*k*|***z***_*i*_,*Y*_*i*1_=1) with *k*=2,3,4.

Let, as before, *T*_1*i*_ be the time to clinically-recognized pregnancy, and now take *C*_*i*_ to be the time to censoring or withdrawal from the first stage of Fig. 1. As a result, we do not necessarily observe *Y*_*i*1_=*I*(*T*_1*i*_≤*τ*) directly, but rather observe (*U*_*i*_,*δ*_*i*_), where *U*_*i*_=(*T*_1*i*_∧*C*_*i*_)∧*τ*,*δ*_*i*_=*I*[*T*_1*i*_≤(*C*_*i*_∧*τ*)], and “ ∧” denotes the minimum of its arguments. Note that *δ*_*i*_≡*Y*_*i*1_ for all individuals with *R*_*i*1_=1; for all remaining individuals, we have only the partial information that *T*_1*i*_>*C*_*i*_. To leverage this when estimating *π*_*i*0_(*τ*), we additionally assume that *T*_1*i*_ ⊥⊥ *C*_*i*_|***x***_*i*_ and replace the logistic regression model in (2) with the discrete-time survival model

6 $$ \begin{aligned} \text{logit}\left[P\left(T_{1i} = t | T_{1i} \geq t, \boldsymbol{x}_{i}\right)\right] = g_{t} + \boldsymbol{\gamma}^{T}\boldsymbol{x}_{i}, \quad t = 1, 2, \ldots, \tau \end{aligned}  $$

where *P*(*T*_1*i*_=*t*|*T*_1*i*_≥*t*,***x***_*i*_) is the transition intensity function *λ*_01_(*t*) under a discrete-time representation of the conception process.

To fit model (6), let *δ*_*it*_=*I*(*δ*_*i*_=1∩*U*_*i*_=*t*). Then after rearrangement, the log-likelihood of ***g***=(*g*_1_,…,*g*_*τ*_) and ***γ*** may be written as

$$\begin{array}{*{20}l} &\log L(\boldsymbol{g}, \boldsymbol{\gamma}|\boldsymbol{U}, \boldsymbol{\delta}, \boldsymbol{x})\\ &= \sum_{i=1}^{n}\sum_{t=1}^{u_{i}}\delta_{it}\text{logit}\left[P\left(T_{1i} = t|T_{1i} \geq t, \boldsymbol{x}_{i}\right)\right] \\ &\quad+ \sum_{i=1}^{n}\sum_{t=1}^{u_{i}}\log\left[1-P\left(T_{1i} = t | T_{1i} \geq t, \boldsymbol{x}_{i}\right)\right]\\ &=\sum_{i=1}^{n}\sum_{t=1}^{u_{i}}\left\{\delta_{it}\!\left(g_{t} \!+ \boldsymbol{\!\gamma}^{T}\boldsymbol{x}_{i}\right) \,-\, \log\!\left[1 \,+\, \exp\!\left(g_{t} \,+\, \boldsymbol{\gamma}^{T}\boldsymbol{x}_{i}\right)\right]\right\}, \end{array} $$

which is the log-likelihood for a logistic regression model fit to $N=\sum _{i=1}^{n}U_{i}$ independent observations with *δ*_*it*_ as the outcome [31]. Estimation of ***g*** and ***γ*** then proceeds by:

1. Creating *U*_*i*_ duplicates of individual *i*’s record, with each duplicate corresponding to a distinct unit of time during which individual *i* was under observation.
2. Recording, for each unit of time, the binary outcome *δ*_*it*_. By definition, *δ*_*it*_=0 for observation times *t*=1,…,*U*_*i*_−1. If individual *i* was censored, then $\delta _{iU_{i}} = 0$ as well; otherwise, $\delta _{iU_{I}} = 1$.
3. Using standard maximum likelihood estimation to fit the logistic regression model logit{*P*(*δ*_*it*_=1|***x***_*i*_)}=*g*_*t*_=***γ***^*T*^***x***_*i*_ under working independence.

We may then recover $\widehat {\pi }_{i\neg 0}(\tau) = \widehat {P}(Y_{i1} = 1 | \boldsymbol {x}_{i}) = \widehat {P}(T_{1i} \leq \tau | \boldsymbol {x}_{i})$ by taking

$$\begin{aligned}\widehat{P}(T_{1i} \leq \tau | \boldsymbol{x}_{i}) &= \sum_{t=1}^{\tau}\left[\widehat{P}(T_{1i} = t | T_{1i} \geq t, \boldsymbol{x}_{i})\right. \\ &\left.\quad\times \prod_{j=1}^{t-1}\left\{1-\widehat{P}(T_{1i} = t-j|T_{1i} \geq t-j, \boldsymbol{x}_{i})\right\}\right] \\ &=\sum_{t=1}^{\tau}\left[\text{expit}\left(\widehat{g}_{t} + \widehat{\boldsymbol{\gamma}}^{T}\boldsymbol{x}_{i}\right) \prod_{j=1}^{t-1}\right.\\ &\left.\quad \left\{1- \text{expit}\left(\widehat{g}_{t-j} + \widehat{\boldsymbol{\gamma}}^{T}\boldsymbol{x}_{i}\right)\right\}{\vphantom{\prod_{j=1}^{t-1}}}\right]. \end{aligned} $$

Estimation of the remaining components of the personalized risk profile requires fitting model (3), which in turn requires addressing missingness in both the selection event, *Y*_*i*1_, and the outcome, *Y*_*i*2_. To do so, we modularize the data provenance for ***Y***_*i*_ into two submechanisms—one corresponding to whether or not the implantation event (or lack thereof) is observed within *τ* menstrual cycles, and the other corresponding to whether or not the final clinical result of that pregnancy is recorded—and use either multiple imputation or inverse probability of censoring weights to address missingness at each of these stages.

In the sequential multiple imputation analysis of (3), we start by imputing the selection event *I*(*T*_1*i*_≤*τ*), which is necessary for delineating the subgroup on whom model (3) will subsequently be fit. This selection event is, in turn, a function of the time to clinically-recognized pregnancy, *T*_1*i*_. Thus, to fit model (3) in the presence of two-stage outcome missingnenss, we first impute *T*_1*i*_ for all individuals with *R*_*i*1_=0 using the multinomial model

$$\begin{aligned} \log \left\{\!\frac{P(T_{1i} = t | T_{1i} > u_{i}, \boldsymbol{x}_{i}^{*})}{P(T_{1i} = u_{i} + 1 | T_{1i} > u_{i}, \boldsymbol{x}_{i}^{*})}\!\right\} \,=\, \boldsymbol{\zeta}_{u_{i}}^{T}\boldsymbol{x}_{i}^{*}, \quad t \,=\, u_{i} \,+\, 1, \ldots, \tau \,+\, 1, \end{aligned} $$

where $\boldsymbol {x}_{i}^{*} \subset \boldsymbol {w}_{i}$ comprises both the predictors ***x***_*i*_ and any other auxiliary variables thought helpful for the imputation process (such as ***x****i*′; see [32] for guidance on specifying the imputation model) and where the mass at cycle *t*=*τ*+1 corresponds to administrative censoring at time *τ*. We then transform the imputed $\widetilde {T}_{1i}$ to obtain the corresponding indicator of an implantation event, $\widetilde {Y}_{i1} = I(\widetilde {T}_{1i} \leq \tau)$. For all individuals meeting the selection criteria for model (3) (i.e., with either *Y*_*i*1_=1 or $\widetilde {Y}_{i1} = 1$) but with missing second-stage outcome data (i.e., with *R*_*i*2_=0), we also conduct a second imputation for the final pregnancy result, $\widetilde {Y}_{i2}$. We repeat this two-stage imputation procedure *M* times, and in the *m*th completed dataset fit model (3) to all individuals with either an observed or imputed implantation event within *τ* menstrual cycles. Combining the resulting point estimates, we find $\widehat {\boldsymbol {\alpha }} = M^{-1}\sum _{m=1}^{M}\widehat {\boldsymbol {\alpha }}^{(m)}$. Alternatively, in the two-stage inverse-probability-weighted analysis of model (3), we first estimate the probability of having complete data with respect to model (3),

$$\begin{array}{*{20}l} \pi_{i}^{C} & =P(R_{i2}=1, Y_{i1} = 1, R_{i1} = 1 | \boldsymbol{w}_{i}) \\ &=P(R_{i1} = 1 | \boldsymbol{z}_{i}', R_{i1} = 1, Y_{i1} = 1) \\ &\quad\ P(Y_{i1} = 1 | \boldsymbol{x}_{i}, R_{i1}= 1)P(R_{i1} = 1 | \boldsymbol{x}_{i}'), \end{array} $$

using (for example) a series of logistic regression models, and arrive at $\widehat {\boldsymbol {\alpha }}$ by fitting (3) to the complete cases, weighted by $1/\widehat {\pi }_{i}^{C}$. Recent work has also considered the task of model estimation and inference when different missing data techniques are adopted for each of the proposed sub-mechanisms [33, 34]. Regardless of how final point estimates for (3) are obtained, given the resulting $\widehat {\boldsymbol {\alpha }}$ we may then estimate $\widehat {\pi }_{i2|1}(\tau), \widehat {\pi }_{i3|1}(\tau)$, and $\widehat {\pi }_{i4|1}(\tau)$ as in the previous section.

##### **Remark 1**

For a given data provenance sub-mechanism, the choice between conducting multiple imputation and conducting inverse probability weighting should largely be informed by which model the analyst feels better able to specify correctly: the imputation model or the weighting model. The former requires modeling the full conditional distribution of the missing data given the observed data—which (at a minimum) draws upon knowledge of the outcome process, and which can become challenging if additional covariates beyond the outcome are missing—while the latter requires modeling *P*(*R*_1_=1) and draws upon knowledge of the missingness process. Misspecification of these models will, in general, lead to bias in the point estimates $\widehat {\boldsymbol {\alpha }}$ from the corresponding imputed or weighted final analysis, though iterative multiple imputation procedures such as multiple imputation by chained equations have been shown to be robust to misspecification of the full conditional distribution so long as the component univariate conditional distributions are valid [35]. Other considerations for selecting a missing data approach include efficiency (when the imputation and weighting models are both correctly specified, multiple imputation is known to be more efficient than inverse probability weighting) and the stability of the inverse probability weights (in settings where either $\widehat {P}(R_{i1} = 1|\boldsymbol {x}_{i}')$ or $\widehat {P}(R_{i2} = 1 | \boldsymbol {z}_{i}', R_{i1}=1, Y_{i1})$ is near zero for certain levels of ***x****i*′ or ***z****i*′, the corresponding inverse probability weights are large and the resulting estimator may be unstable).

We must also account for both partial and complete missingness in ***Y***_*i*_=(*Y*_*i*1_,*Y*_*i*2_) when quantifying the discriminatory performance of the predicted risk profiles, $\widehat {p}_{i}(\tau)$. In particular, the nonparametric HUM estimator given in (5) requires knowledge of each individual’s true pregnancy outcome; a complete-case implementation—in which (5) is estimated using only the subset of individuals for whom this outcome is completely observed—may be subject to verification bias unless both *Y*_*i*1_ and *Y*_*i*2_ are missing completely at random. Zhang and Alonzo [36] consider verification bias adjustment in the three outcome classification setting in which the outcomes of interest are ordinal and the classification decision is based on a continuous measurement; under the additional assumption that missingness occurs at random, they develop an inverse-probability-weighted version of the nonparametric HUM estimator. Here, we extend this result to *K*=4 unordered outcome classes, missing sequentially at random, where classification occurs on the basis of a multinomial risk profile.

Let *R*_*i*1_,*R*_*i*2_, and *R*_*i*_ be defined as before, and let $n_{k}^{*}$ be the number of individuals in outcome class *k* with *R*_*i*_=1. Then a verification-bias-adjusted estimator for the HUM is

7 $$ \frac{\sum_{i_{1}=1}^{n_{1}^{*}}\sum_{i_{2}=1}^{n_{2}^{*}}\sum_{i_{3}=1}^{n_{3}^{*}}\sum_{i_{4}=1}^{n_{4}^{*}} (\widehat{\pi}_{i_{1}}^{R} \widehat{\pi}_{i_{2}}^{R}\widehat{\pi}_{i_{3}}^{R}\widehat{\pi}_{i_{4}}^{R})^{-1}CR\left(\widehat{p}_{i_{1}}^{(1)}(\tau), \widehat{p}_{i_{2}}^{(2)}(\tau), \widehat{p}_{i_{3}}^{(3)}(\tau), \widehat{p}_{i_{4}}^{(4)}(\tau)\right)}{\sum_{i_{1}=1}^{n_{1}^{*}}\sum_{i_{2}=1}^{n_{2}^{*}}\sum_{i_{3}=1}^{n_{3}^{*}}\sum_{i_{4}=1}^{n_{4}^{*}}(\widehat{\pi}_{i_{1}}^{R}\widehat{\pi}_{i_{2}}^{R}\widehat{\pi}_{i_{3}}^{R}\widehat{\pi}_{i_{4}}^{R})^{-1}},  $$

where $\widehat {p}_{i_{k}}^{(k)}(\tau)$ is the estimated risk profile for the *i*_*k*_th individual in outcome class *k*, and where $\widehat {\pi }_{i_{k}}^{R}$ is the estimated probability of fully observing ***Y***_*i*_ for that same individual:

$$\widehat{\pi}_{i}^{R} = \widehat{P}\left(R_{i2} = 1 | \boldsymbol{z}_{i}', R_{i1} = 1, Y_{i1}\right)\widehat{P}\left(R_{i1} = 1 | \boldsymbol{x}_{i}^{\prime}\right). $$

### Preconception risk prediction in the EAGeR study population

We illustrate the broad utility of this multistate competing risks framework for pregnancy outcomes by constructing and validating a preconception risk assessment tool on the study population of the Effects of Aspirin in Gestation and Reproduction (EAGeR) trial [23, 37]. As discussed in the “Background” section, the trial enrolled 1228 women who were actively attempting to conceive, and who were between the ages of 18 and 40, had a history of 1–2 previous pregnancy losses, had up to two previous live births, and had at most one elective termination or ectopic pregnancy; hese women had no prior history of infertility or subfertility, and were neither currently undergoing nor planning to undergo medical fertility treatment during the course of the trial. Each woman attended a baseline preconception clinic visit, at which point biomedical, sociodemographic, and medical data were collected. The women were then randomized to take either low-dose aspirin or a placebo over the remainder of their pregnancy attempt. The women were followed for up to six menstrual cycles for the occurrence of a clinically-recognized pregnancy and—for those women who successfully conceived during the study period—were subsequently followed for the outcome of that pregnancy (Table 1).

**Table 1:** Observed pregnancy outcomes for the *n*=1093 EAGeR participants who were not clinically infertile at the baseline preconception visit

Pregnancy Outcome^a^	*R*	*Y*	*N* (%)
Censored prior to conception	(*R*_1_=0,*R*_2_=0)	—	88 (8.1%)
No pregnancy within 6 cycles	(*R*_1_=1,*R*_2_=1)	*Y*_1_=0	319 (29.2%)
Pregnancy within 6 cycles			
Unknown result	(*R*_1_=1,*R*_2_=0)	(*Y*_1_=1, —)	12 (1.1%)
Clinical pregnancy loss	(*R*_1_=1,*R*_2_=1)	(*Y*_1_=1,*Y*_2_=2)	122 (11.2%)
Preterm birth	(*R*_1_=1,*R*_2_=1)	(*Y*_1_=1,*Y*_2_=3)	49 (4.5%)
Full-term birth	(*R*_1_=1,*R*_2_=1)	(*Y*_1_=1,*Y*_2_=4)	501 (45.8%)

^a^A pregnancy was considered to be clinically-recognized if it was confirmed by an ultrasound scan at 6–7 weeks’ gestation. Clinical pregnancy loss comprised both miscarriages (prior to 20 weeks’ gestation) and stillbirths (after 20 weeks’ gestation), preterm birth comprised live births prior to 37 weeks’ gestation, and full-term birth comprised live births after 37 weeks’ gestation

Towards building a risk prediction model for the outcome of these pregnancy attempts given a conception window of *τ*=6 menstrual cycles, we restricted our attention to the 1093 women who had been attempting to conceive for fewer than twelve menstrual cycles at the start of the trial and who contributed at least one cycle of follow-up. We considered the following covariates as possible predictors, ***w***_*i*_: preconception aspirin use (0, placebo; 1, low-dose aspirin), age at enrollment, body mass index (BMI), hypertensive status (0, non-hypertensive; 1, hypertensive), smoking history over the past year (0, none; 1, at least one cigarette), race (0, other; 1, non-Hispanic white), income level (≤ $19,999; $20,000–$39,999; $40,000–$74,999; $75,000–$99,999; ≥$100,000), educational attainment (0, some college or less; 1, college or postgraduate degree), parity (0, nulliparous; 1, parous), number of previous pregnancy losses (either 1 or 2 per study inclusion criteria), and number of menstrual cycles spent attempting to conceive at the time of enrollment.

#### Implementation of the proposed model and validation metric

Given that women in the EAGeR trial reported missingness at both stages of the pregnancy outcome process (Table 1), as well as at low levels in the available baseline covariate data (BMI, 1.4%; hypertensive status, 0.5%; smoking status, 0.7%; educational attainment, 0.7%), we estimated and validated the personalized risk profiles according to the procedure proposed in the “Estimation and validation in the presence of outcome missingness” section. In particular, we conducted multiple imputation by chained equations to create *M*=10 imputed datasets: for the first-stage model in (6), we imputed missing baseline covariate data only, while for the second-stage model in (3), we also used a sequential multiple imputation procedure to impute both missing selection events and missing pregnancy outcome data. We then selected predictors separately for the first- and second-stage models using an AIC-based backward selection procedure applied to the combined dataset of *Mn* individuals in order to decide among all possible first-order covariate effects [38]. Both race and number of previous losses were a priori thought to be clinically relevant, and so were manually included in both stages of the outcome model. We fit the selected models on each imputed dataset separately—with the first-stage model fit using all fertile women in the EAGeR trial, and the second-stage model fit using all women with either an observed or imputed clinically-recognized pregnancy—and combined the point estimates across the datasets using the standard Rubin’s method.

To quantify the predictive capability of the final pregnancy outcome model, we implemented the verification-bias-adjusted HUM estimator in (7). In light of the low levels of second-stage outcome missingness (only 12 women had recognized pregnancies but no recorded final pregnancy outcome), we fit a single logistic regression model to estimate the probability of verification weights, $\widehat {\pi }_{i}^{R} = \widehat {P}(R_{i} = 1 | \boldsymbol {w}_{i})$, and once again used backward selection followed by Rubin’s method to arrive at the final model for the verification weights.

Finally, to adjust for the optimistic prediction performance inherent to both fitting and validating models (6) and (3) on the full EAGeR dataset, we implemented the optimism correction described in Harrell et al. [39]. Let $\widehat {HUM}_{init}$ be the initial optimistic verification-bias-adjusted HUM estimate. We first created *B*=1000 bootstrapped datasets, and in each of these datasets repeated the multiple imputation, model selection, and model estimation procedures in order to refit models (6) and (3) and reestimate the verification weights, $\widehat {\pi }_{i}^{R}$ [40]. For the *b*th set of bootstrapped models, we then evaluated their prediction performance (i.e., computed the verification-bias-adjusted HUM estimator) on both the corresponding bootstrapped dataset and the original EAGeR dataset: $\widehat {HUM}_{boot}^{(b)}$ and $\widehat {HUM}_{orig}^{(b)}$, respectively. The final optimism-corrected estimate was then

$$\widehat{HUM} = \widehat{HUM}_{init} - \frac{1}{B}\sum_{b=1}^{B}\left(\widehat{HUM}_{boot}^{(b)} - \widehat{HUM}_{orig}^{(b)}\right). $$

##### **Remark 2**

The above bootstrap procedure explicitly accounts for three sources of uncertainty and optimism in $\widehat {HUM}_{init}$, namely that resulting from the multiple imputation, the model selection, and the model estimation procedures. However, several of the covariate patterns in ***w***_*i*_ have low prevalence in the original and resampled EAGeR datasets, such that the final selected models were highly variable across the resampled datasets and prone to large and unstable coefficient estimates; these large fluctuations in coefficient magnitude in turn led to increased separation between the individual risk predictions and thus to improved in-sample (and worsened out-of-sample) discriminatory performance (a model selection analog of the *winner’s curse* [41]). As a result, the resampled $\widehat {HUM}_{boot}$ were overly optimistic relative to $\widehat {HUM}_{init}$, and their empirical bootstrap distribution was centered well to the right of the observed-sample statistic. In light of this idiosyncratic small-sample behavior, we opt to report bootstrapped optimism-corrected results that condition on the form of the originally-selected risk prediction model, i.e., for which only the multiple imputation and model estimation procedures were bootstrapped.

All analyses were conducted using R version 3.6.1, and the code is available online at https://github.com/kaitlyncook/preconception-risk-prediction. The analysis code makes use of the package discSurv to fit the discrete-time survival model in (6), nnet to fit the multinomial regression model in (3), MASS to conduct model selection, and mice to conduct sequential multiple imputation.

## Results

**Fig. 3:**
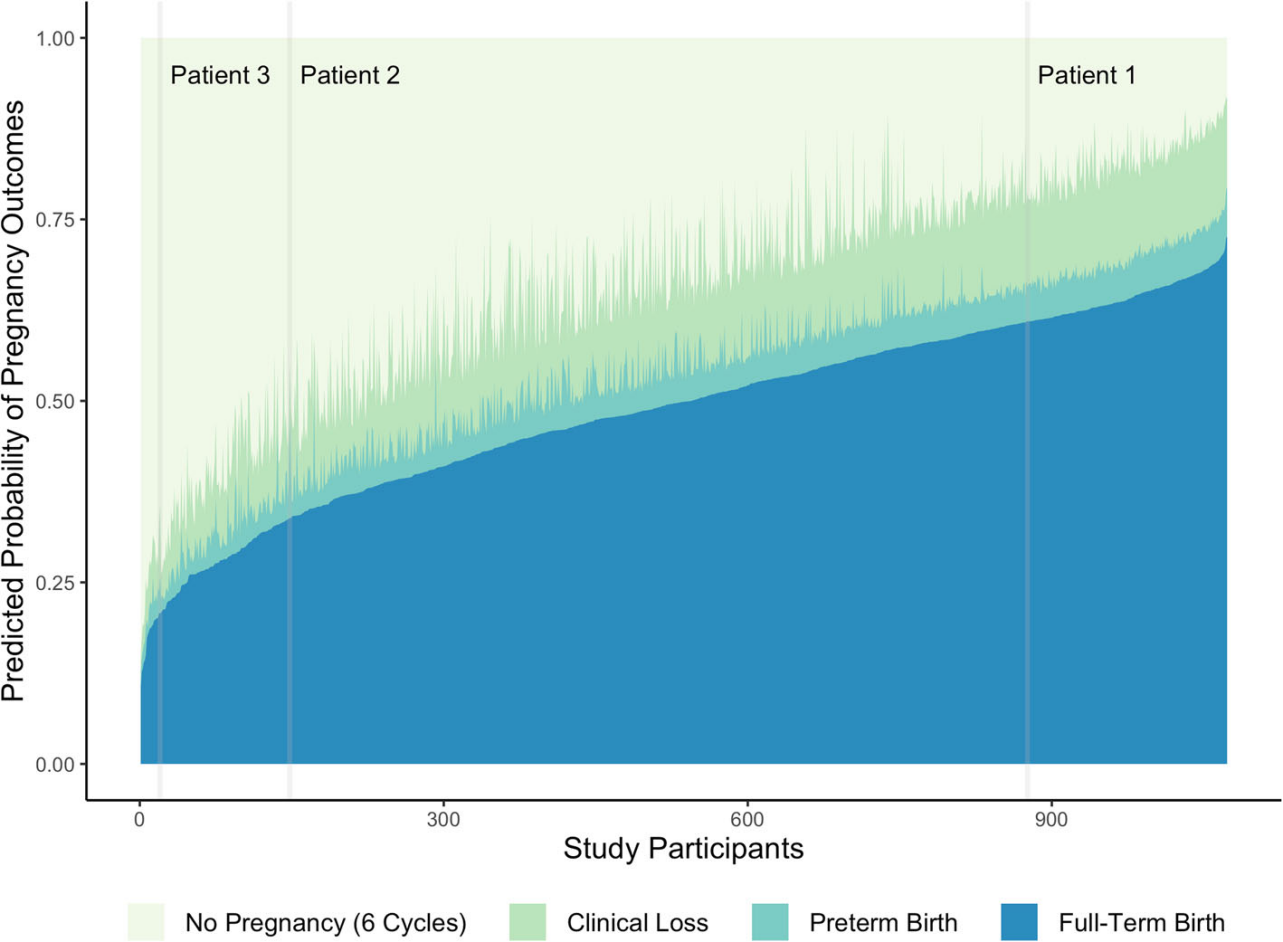
Preconception risk profiles for all *n*=1073 women in the EAGeR trial with complete baseline covariate data with respect to the final prediction models. Each vertical cross-section of the plot corresponds to a unique individual in the EAGeR trial; for each of these women, the height of each colored region represents her predicted probability of the corresponding pregnancy outcome. The gray vertical bars correspond to the predicted risk profiles of the three patients in Table 3

**Fig. 4:**
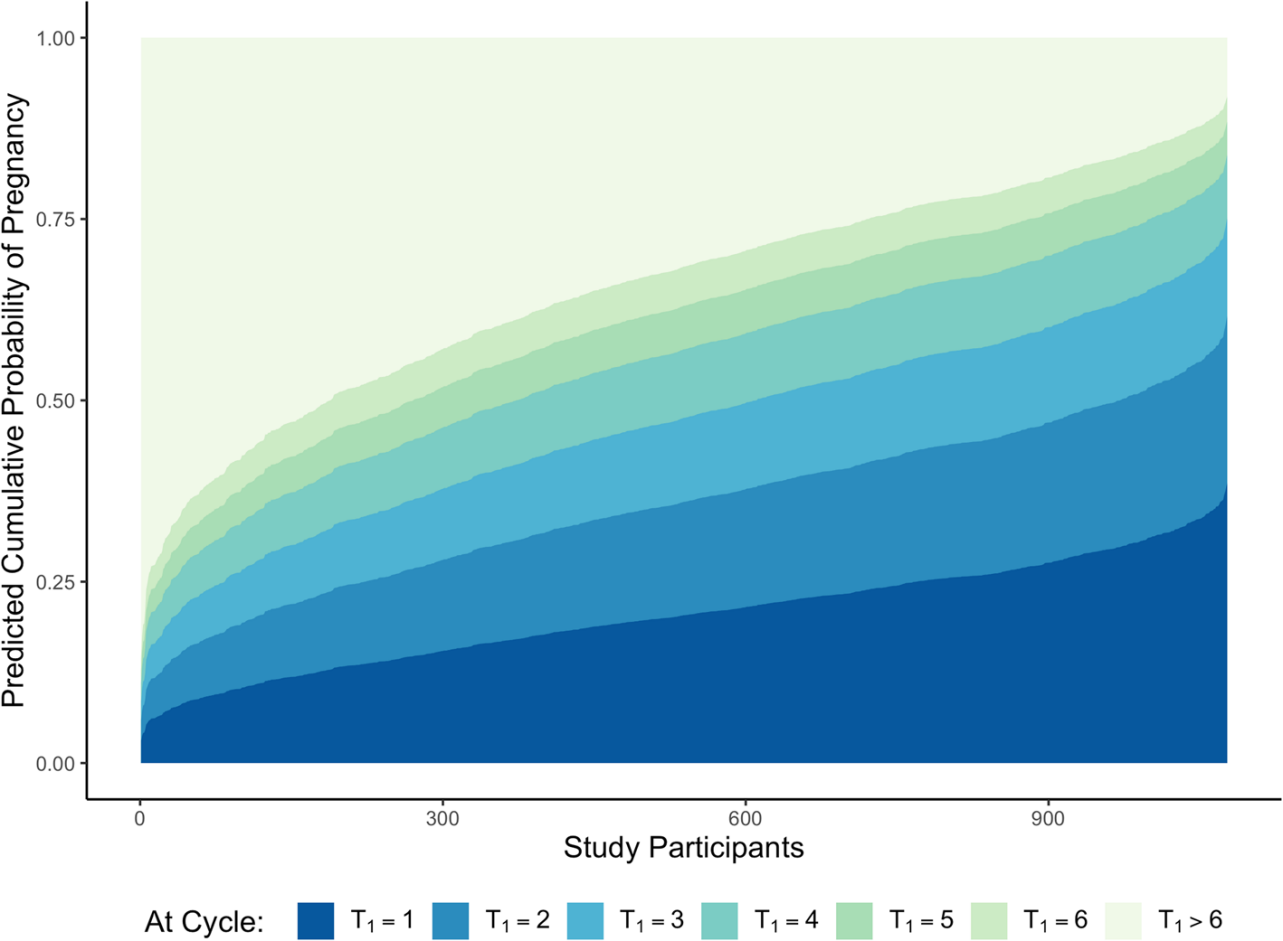
Probability of the first clinically-recognized pregnancy occurring at *T*_1_ menstrual cycles post preconception visit for all *n*=1073 women in the EAGeR trial with complete baseline covariate data with respect to the final prediction models. Each vertical cross-section of the plot corresponds to a unique individual in the EAGeR trial; for each of these women, the height of each colored region represents her predicted probability of conceiving during the corresponding menstrual cycle

The final first- and second-stage prediction models are given in Table 2, and the resulting preconception risk profiles, calculated for all women in the EAGeR trial with complete covariate data, are given in Fig. 3. Among these women, the predicted probability of a clinically-recognized pregnancy within the first six menstrual cycles post preconception visit ranged from 14.5% to 91.9%; the predicted probability of successful conception peaked during the first menstrual cycle post clinic visit (at 20.3%, on average) and decreased with each subsequent cycle (Fig. 4). Assuming that each woman did, in fact, successfully conceive, the most likely outcome of their pregnancies was a full-term birth (with predicted conditional probabilities ranging from 56.7% to 80.2%), followed by clinical pregnancy loss (10.1% to 37.0%) and preterm birth (3.5% to 25.7%). The optimistic verification-bias-adjusted HUM was estimated to be 0.108 (95% confidence interval (CI) with bootstrapped standard errors: 0.078, 0.144), while the optimism-corrected HUM was estimated to be 0.093 (95% CI with bootstrapped standard errors: 0.064, 0.128); both point estimates and confidence interval limits fell well above the non-informative HUM for a four-level outcome (≈0.042), representing significant improvement over classification by random chance.

**Table 2:** Selected models for the time to clinically-recognized pregnancy (first-stage model), the result of that pregnancy (second-stage model) and the probability of verification. All results are reported as: estimate (SD)

	First Stage	Second Stage		Verification
	logit[*P*(*T*_*i*_=*t*|*T*_*i*_≥*t*)]	log(*π*_*i*2|1_/*π*_*i*4|1_)	log(*π*_*i*3|1_/*π*_*i*4|1_)	$\text {logit}\left (\pi _{i}^{R}\right)$
Intercept	-0.542 (0.370)	-3.025 (0.754)	-0.827 (1.096)	2.050 (0.515)
*I*(*t*=2)	-0.047 (0.117)	—	—	—
*I*(*t*=3)	-0.148 (0.129)	—	—	—
*I*(*t*=4)	-0.152 (0.141)	—	—	—
*I*(*t*=5)	-0.456 (0.167)	—	—	—
*I*(*t*=6)	-0.402 (0.178)	—	—	—
Menstrual cycles trying prior to baseline	-0.120 (0.019)	—	—	—
Aspirin use	0.185 (0.086)	—	—	—
Age (in years)	-0.026 (0.010)	0.041 (0.022)	-0.032 (0.035)	—
Number of previous pregnancy losses	0.058 (0.092)	0.265 (0.210)	-0.089 (0.330)	—
Non-Hispanic white	0.444 (0.159)	0.012 (0.422)	-0.698 (0.463)	0.773 (0.278)
College degree	0.251 (0.093)	—	—	0.474 (0.236)
BMI	-0.027 (0.007)	—	—	-0.024 (0.015)
Parous	0.357 (0.090)	—	—	0.366 (0.214)
Smoker (past year)	—	—	—	-0.973 (0.258)
Hypertension	—	0.412 (0.253)	0.741 (0.367)	—

**Table 3:** Preconception medical history and sociodemographic profiles for three hypothetical patients, along with their corresponding predicted preconception risk profiles

	Patient 1	Patient 2	Patient 3
*Characteristics at the Preconception Visit*			
Age	28.3 years	23.7 years	39.3 years
Non-Hispanic white	Yes	No	No
College degree	None	None	None
BMI	24.4	21.1	27.2
Hypertensive status	No	Yes	Yes
Parous	Yes	No	Yes
Number of previous pregnancy losses	1.0	1.0	1.0
Preconception aspirin use	Yes	No	No
Menstrual cycles trying prior to visit	1.0	2.0	4.0
*Preconception Risk Profile*			
No pregnancy within *τ*=6 cycles	0.212	0.443	0.643
Pregnancy ending in clinical loss	0.125	0.085	0.099
Pregnancy ending in a preterm birth	0.050	0.134	0.050
Pregnancy ending in a full-term birth	0.613	0.338	0.208

To illustrate the potential clinical utility of this multistage prediction framework, we specifically highlight the predicted risk profiles for three hypothetical patients: one who represents the median participant in the EAGeR trial with respect to each of the included baseline covariates; another whose medical history, and in particular their hypertension and experience of previous pregnancy loss with no previous live birth, places them at higher risk of an adverse pregnancy outcome; and a third who is of advanced maternal age (Table 3). Comparing the corresponding risk profiles to one another illustrates a stark difference in predicted pregnancy attempt trajectories for the three hypothetical patients, which may then inform the subsequent preconception care that each patient receives (see the gray bands superimposed on Fig. 3). The first patient and their obstetrician may, for example, find their preconception risk profile to be acceptable, and proceed with the pregnancy attempt according to the standard of care. The second patient, however, has an elevated probability of failing to conceive (44.2%) and, should they conceive, of having either a clinical pregnancy loss or preterm birth (22.0%). Given this profile, the obstetrician may instead recommend more active monitoring of the pregnancy attempt and, should the patient fail to conceive within six additional menstrual cycles, a fertility treatment such as intrauterine insemination. Finally, the third patient’s advanced maternal age and high risk of either failing to conceive (64.3%) or experiencing a clinical pregnancy loss (9.9%) might lead the obstetrician to recommend immediate use of assisted reproductive technology.

## Discussion

In this manuscript, we have advocated for the use of a multistate modeling framework in order to construct and validate individualized pregnancy outcome profiles for use in routine preconception care. We have discussed how the task of pregnancy outcome prediction in particular presents challenges to the typical construction and application of these multistate models—namely regarding correct specification of the transition intensities and potential coarsening of the available data on both the outcome and censoring processes—and presented a two-stage estimation process that circumvents these challenges in order to derive patient-specific absolute risk profiles for the joint probability of all competing birth outcomes. We also developed an inverse-probability-weighted HUM statistic to quantify the model’s classification performance.

By adopting this multistate prediction framework, we were able to reframe—and broaden the purview of—pregnancy risk prediction to include endpoints related to both the conception and gestation processes. Existing prediction tools have typically focused on a single adverse pregnancy outcome, whose risk is determined on the basis of biomedical markers collected over the course of the pregnancy; they are necessarily calculated downstream of the initial preconception consultation. This initial visit represents, however, an important window in patients’ preconception and prenatal care. It marks the first time at which risk factors for adverse conception and perinatal outcomes may be identified and modified, with potentially long-ranging implications for the pregnancy attempt. To that end, the multistate competing risks framework allows one to derive patient-specific risk profiles—estimable at the time of the preconception visit—that quantify both (i) the likelihood of successful conception and (ii) the likelihood of that pregnancy ending in a clinical pregnancy loss, preterm birth, or full-term birth while permitting different sets of demographic and medical characteristics to govern and inform each of those processes.

We illustrated the clinical utility of these risk profiles by constructing and validating a multistage competing risks model using the EAGeR trial. While the patient population in the EAGeR trial—women with at least one previous pregnancy loss and at most two previous live births—prevents generalization of this prediction model to a broader clinical population, it still represents an important proof of concept. Despite using only the (more limited) information available at the time of the initial preconception visit, the resulting risk profiles were able to meaningfully distinguish between women on the basis of their future pregnancy outcomes. This prediction framework also easily generalizes to other clinical settings in which the endpoint of interest is effectively a multistage outcome. For example, predicting which patients are at high-risk of hospital-acquired infections and subsequent adverse events—which considers first whether a patient acquires an infection and, among those with a hospital-acquired infection, the duration of stay or severity of that infection—readily lends itself to a similar multistage framing.

Before these sorts of multistate competing risks prediction models are implemented in clinical practice, however, several limitations regarding their construction and validation must be addressed. Returning to the task of pregnancy outcome prediction, some fraction of the broader population is understood to be *sterile* and thus unable to conceive or to experience any of the downstream pregnancy outcomes; this phenomenon is naturally modeled through the inclusion of either a latent sterility variable or a so-called cure fraction (here representing those who are sterile) into the competing risks model. These extensions are not without their own methodological challenges. An individual’s latent sterility status is measured imperfectly through their observed *fertility*, which itself is measured only after a fixed period of time—an individual is classified as clinically infertile only after unsuccessfully attempting to conceive for 12 or more menstrual cycles—and so will be structurally missing for most individuals in the estimation and validation datasets. Once measured, sterility is also subject to differential misclassification: while all individuals who are unable to conceive will be labeled as clinically infertile, not all individuals labeled as clinically infertile are biologically unable to conceive. Generalizing the multistate pregnancy outcome prediction framework to include infertility will thus require addressing both of these statistical features. Additional work will also be needed to improve model selection procedures for multistate competing risks models, e.g., through the development of penalized regression methods, particularly when these models are fit using either inverse probability of censoring weights or multiple imputation.

Finally, the proposed HUM validation metric for multinomial outcomes is less well-studied and well-known than its binary counterpart, the AUC, and so lacks the same common understanding of what a “poor”, “good”, or “excellent” HUM might be for a given applied problem. Constructing confidence intervals for the estimated HUM is an important first step towards contextualizing the predictive performance of multinomial or nested multinomial (multistate) models, but further research on both the statistical properties and the practical implications of the HUM is warranted.

## Conclusion

As illustrated by our analysis of the EAGeR trial data, our proposed multistate competing risks framework has the potential to shape preconception care by (i) considering joint risk prediction for a broader array of possible pregnancy attempt outcomes and (ii) providing those predictions at an earlier intervention period (during the preconception clinic visit) than existing obstetric prediction models. It thus presents an important first step in expanding the types of clinical risk assessment tools available for pregnancy outcome prediction, and suggests broader applications to the prediction of other complex, multistage health outcomes.

## Data Availability

The data analyzed in this study are available upon request at the NICHD/DIPHR Biospecimen Repository Access and Data Sharing (BRADS) site (https://brads.nichd.nih.gov).

## Abbreviations

EAGeR: Effects of Aspirin in Gestation and Reproduction
HUM: Hypervolume under the manifold
AUC: Area under the curve
BMI: Body mass index
CI: Confidence interval
